# Strong Synergism of Palmatine and Fluconazole/Itraconazole Against Planktonic and Biofilm Cells of *Candida* Species and Efflux-Associated Antifungal Mechanism

**DOI:** 10.3389/fmicb.2018.02892

**Published:** 2018-12-03

**Authors:** Tianming Wang, Jing Shao, Wenyue Da, Qianqian Li, Gaoxiang Shi, Daqiang Wu, Changzhong Wang

**Affiliations:** ^1^Laboratory of Biochemistry and Molecular Biology, College of Integrated Chinese and Western Medicine (College of Life Science), Anhui University of Chinese Medicine, Hefei, China; ^2^Laboratory of Pathogenic Biology and Immunology, College of Integrated Chinese and Western Medicine (College of Life Science), Anhui University of Chinese Medicine, Hefei, China

**Keywords:** palmatine, fluconazole, itraconazole, *Candida* species, efflux, resistance

## Abstract

Fungal infections caused by *Candida albicans* and non-*albicans Candida* [NAC] species are becoming a growing threat in immunodeficient population, people with long-term antibiotic treatment and patients enduring kinds of catheter intervention. The resistance to one or more than one conventional antifungal agents contributes greatly to the widespread propagation of *Candida* infections. The severity of fungal infection requires the discovery of novel antimycotics and the extensive application of combination strategy. In this study, a group of *Candida* standard and clinical strains including *C. albicans* as well as several NAC species were employed to evaluate the antifungal potentials of palmatine (PAL) alone and in combination with fluconazole (FLC)/itraconazole (ITR) by microdilution method, checkerboard assay, gram staining, spot assay, and rhodamine 6G efflux test. Subsequently, the expressions of transporter-related genes, namely *CDR1*, *CDR2*, *MDR1*, and *FLU1* for *C. albicans*, *CDR1* and *MDR1* for *Candida tropicalis* and *Candida parapsilosis*, *ABC1* and *ABC2* for *Candida krusei*, *CDR1*, *CDR2*, and *SNQ2* for *Candida glabrata* were analyzed by qRT-PCR. The susceptibility test showed that PAL presented strong synergism with FLC and ITR with fractional inhibitory concentration index (FICI) in a range of 0.0049–0.75 for PAL+FLC and 0.0059–0.3125 for PAL+ITR in planktonic cells, 0.125–0.375 for PAL+FLC and 0.0938–0.3125 for PAL+ITR in biofilms. The susceptibility results were also confirmed by gram staining and spot assay. After combinations, a vast quantity of rhodamine 6G could not be pumped out as considerably intracellular red fluorescence was accumulated. Meanwhile, the expressions of efflux-associated genes were evaluated and presented varying degrees of inhibition. These results indicated that PAL was a decent antifungal synergist to promote the antifungal efficacy of azoles (such as FLC and ITR), and the underlying antifungal mechanism might be linked with the inhibition of efflux pumps and the elevation of intracellular drug content.

## Introduction

*Candida*-related infections are becoming a universal threat to the health of human who usually undergo immunosuppressive therapy or aggressive medical intervention. The most commonly found *Candida* species is *C. albicans*, an amicable resident in human intestinal tract and genital mucosal surface which can switch into an opportunistic pathogen when external environmental cues are altered (such as pH, temperature, immunocompetence, etc.) (Poulain, 2013; Sardi et al., 2013). With the expanding spread of antifungal agents and the frequent use of broad-spectrum immune-inhibitors, emerging non-*albicans Candida* (NAC) species including *C. glabrata*, *C. parapsilosis*, *C. tropicalis*, *C. krusei* and *C. guilliermondii* are causing increasingly high morbidity and mortality. Besides *C. albicans* accounting for most cases of invasive candidosis (ca. 35–55%) (Messer et al., 2006), the above-mentioned NAC are also capable to cause a series of disturbs ranging from mildly superficial mucosal discomforts to deadly disseminated bloodstream and deep-seated tissue infections. The candidosis incidence attributed to NAC species are reported to be varying in a range of 14–21% for *C. glabrata*, 15–23% *C. parapsilosis*, 20–45% for *C. tropicalis*, 2.7% for *C. krusei*, and 0.6–3.7% for *C. guilliermondii* depending on infection site and geography (Pfaller et al., 2006, 2014; Savini et al., 2011; Silva et al., 2012; Rodrigues et al., 2014).

The relatively high incidence rates of *C. albicans* and NAC species are supposed to be closely related with their recalcitrant resistance to conventional antifungal agents including, for example, azoles, polyenes, echinocandins and the unique phenotype biofilms (Silva et al., 2012; Zavrel and White, 2015). Fluconazole (FLC) and itraconazole (ITR) are two commonly prescribed antifungal drugs which exert decent inhibitions on most pathogenic *Candida* species by interrupting the biosynthesis of the fungal-specific membrane sterol ergosterol. However, *C. glabrata*, *C. krusei*, and *C. guilliermondii* have been revealed to be inherently resistant to azoles (Sanguinetti et al., 2015). Although less primary resistances to FLC are reported in *C. albicans* (1.4%), *C. parapsilosis* (3.6%) and *C. tropicalis* (4.1%), it should be noted that an ascending tendency of acquired resistance to azoles are emerging in the three *Candida* species which have been recorded to take up 48% of resistant isolates in a retrospective case-comparator study (Oxman et al., 2010; Pfaller et al., 2010b). Of increasing concern are the frequency of multidrug resistant isolates of *C. albicans* and NAC which display cross-resistance to azoles in especially high-risk patients receiving antifungal prophylaxis (Pfaller et al., 2010a).

Facing increasing severity of global resistance of *Candida* species recovered clinically, the lag of effective drugs with antifungal purpose alone and/or in combination with traditional antimycotics is worrying. Palmatine (PAL) is an isoquinoline alkaloid of medicinal plant drug that can be isolated from *Rhizoma Coptidis* and *Mahonia aquifolium*. PAL exhibits diverse biochemical and pharmacological functions on jaundice, dysentery, hypertension, inflammation, bacterial, virus and fungal infections with less host toxicity (Vollekova et al., 2003; Ishikawa et al., 2016; Wu et al., 2016). Xiao et al. (2015) reported that PAL in combination with berberine (BER) presented remarkably strong antifungal activities to treat *Microsporum canis*-induced dermatitis in rabbits. Besides, increasing evidence also favored the anti-*Candida* effects of PAL, inferring that PAL might deserve for further reversion study of resistance especially to azoles (such as FLC and ITR) and underlying mechanism, for example, related with efflux pumps.

This study aims to study the synergism of PAL with FLC and ITR against *C. albicans* SC5314 and several FLC-resistant clinical isolates as well as five NAC standard isolates including *C. glabrata*, *C. parapsilosis*, *C. tropicalis*, *C. krusei*, and *C. guilliermondii* by susceptibility test, spot assay and gram staining. The antifungal effects of PAL alone and in combination with FLC or ITR on rhodamine 6G efflux and the expressions of efflux pump genes are also examined.

## Materials and Methods

### Strains and Cultivation

*C. albicans* SC5314 was a gift of Prof. Yuanying Jiang, School of Pharmacy, Second Military Medical University (Shanghai, China). Six clinical *C. albicans* isolates including Z3044, Z2003, Z1402, Z1407, Z826, and Z1309 were donated by Huaiwei Lu, Clinical Laboratory, Anhui Provincial Hospital (Hefei, China). *C. parapsilosis* ATCC22019 and *C. tropicalis* GDM2.147 were purchased from Guangdong Culture Collection Center (Guangzhou, China). *C. glabrata* ATCC2340 and *C. krusei* ATCC1182 were obtained from Bianzhen Biotech. Co. (Nanjing, China). *C. guilliermondii* ATCC6260 were acquired from Shfeng Biotech. Co. (Shanghai, China). All of stock cultures of these strains were routinely maintained in Sabouraud’s agar and were propagated in Liquid Sabouraud Medium (Hope Biotech Co., Qingdao, China) at 37°C for 12–16 h till the strains reached the exponential growth phase. The revived *Candida* cells were pooled by centrifugation of 3000 *g*. After washing twice by sterile phosphate-buffered saline (PBS, Leagene, Beijing, China), the fungal cells were resuspended in RPMI-1640 medium (Invitrogen, Carlsbad, CA, United States) without pH adjustment and adjusted to a defined cell density using a hemocytometer prior for following tests.

### Antifungal Activities of Pal Alone and in Combination With FLC and ITR

#### Planktonic Cells

The initial inoculum was adjusted to 2 × 10^3^ CFU/mL. The minimum inhibitory concentrations (MICs) of Pal, FLC, and ITR were performed in a 96-well flat-bottomed microplate (Corning, Corning, NA, United States) by microdilution method. The concentrations of the tested drugs were serially twofold diluted in a range of 2–1024 μg/mL for Pal, 0.125–1024 μg/mL for FLC and 0.125–512 μg/mL for ITR, respectively. The fungal cells were incubated with the drugs used at 37°C for 48 h. The interaction of two drugs was carried out by checkerboard assay with initial inoculum of 2 × 10^6^ CFU/mL at 37°C for 48 h according to Clinical and Laboratory Standards Institute (CLSI) M27-A3 (CLSI, 2008). The concentrations of the tested drugs were also serially twofold diluted in a range of 0.125–512 μg/mL for Pal, 0.015625–16 μg/mL for FLC and 0.03125–8 μg/mL for ITR, respectively. The MIC_80_ was determined as the drug concentration that inhibited 80% of planktonic fungal cells by comparing the optical density (OD) with that of drug-free control at 630 nm. The fractional inhibitory concentration index (FICI) was calculated as (MIC-PAL in combination/MIC-PAL alone) plus [MIC-FLC (ITR) in combination/MIC-FLC (ITR) alone], in which synergism was interpreted as FICI ≤ 0.5, indifference was defined as 0.5 < FICI < 4.0, and antagonism was FICI ≥ 4.0 (Odds, 2003).

#### Biofilm Cell

The initial fungal cells were set at 2 × 10^6^ CFU/mL. For sessile MIC (SMIC), the tested concentrations ranged from 1 to 1024 μg/mL for PAL, FLC, and ITR. In checkerboard assay, the final concentrations of the three drugs were modified in a range of 0.03125–512 μg/mL. The SMIC_80_ was defined as the drug concentration that inhibited 80% of fungal biofilm cells by comparing the metabolic activity with that of drug-free control at 490 nm using XTT method. The XTT procedures were the same as our previous work (Shao et al., 2015). The sessile fractional inhibitory concentration index (sFICI) was calculated as (SMIC-PAL in combination/SMIC-PAL alone) plus [SMIC-FLC (ITR) in combination/SMIC-FLC (ITR) alone], in which synergism was interpreted as sFICI ≤ 0.5, indifference was defined as 0.5 < sFICI < 4.0, and antagonism was sFICI ≥ 4.0 (Shao et al., 2014).

### Spot Assay

The procedures were described as a previous report with less modifications (Mukhopadhyay et al., 2002). Briefly, the revived fungal cells were resuspended and 10-fold diluted by RPMI-1640 medium to three final concentrations of 1 × 10^2^–1 × 10^4^ CFU/mL. Five microliters of the prepared serial dilutions of each *Candida* culture was spotted onto YPD plates in the absence (control) and presence of PAL, FLC, PAL+FLC at the concentrations showing synergism. Growth differences were recorded following incubation of the plates for 48 h at 37°C.

### Gram Staining

One hundred microliter strain medium (2 × 10^6^ CFU/mL) was co-incubated with the same volumes of PAL and/or FLC at the final concentrations showing synergism in susceptibility tests in a 96-well flat-bottomed microplate at 37°C for 6 h. The supernatant was then discarded, resuspended with 100 μL PBS, stained with Gram solution (Leagene, Beijing, China), observed by oil lens (×1000) and photographed by OLYMPUS IX51 (Tokyo, Japan).

### Rhodamine 6G Efflux

The experiment was performed according to the procedures described in one of our previous work with few modifications (Shao et al., 2016). Briefly, two milliliters of fungal culture was firstly adjusted to 2 × 10^6^ CFU/mL with RPMI 1640, and then co-incubated with the same volume of PAL, FLC, and PAL+FLC at the concentrations showing synergism at 37°C for 24 h. The supernatant was discarded after 2000 *g* of centrifugation. The remnants were mixed with 2 mL sterile PBS for 2 h of incubation at 37°C in an orbital shaker (ca. 200 rpm). The final concentration of 10 μM rhodamine-6G (150 μL) was added for 2 h of incubation at 37°C without light. The supernatant was discarded after 3000 *g* of centrifugation. The pellets were washed three times by sterile PBS. The glucose required for rhodamine 6G efflux was dissolved in PBS to the final concentration of 2 mM. The suspension was centrifuged at 3000 *g*, and 100 μL of supernatant was visualized at the excitation wavelength of 525 nm and the emission wavelength of 550 nm by an inverted fluorescence microscope IX71 (Olympus, Tokyo, Japan).

### Gene Expressions

The procedures of qRT-PCR analysis were described in one of our previous study with small modifications (Shao et al., 2016). Briefly, 1 × 10^6^ CFU/mL of fungal culture was incubated for 24 h at 37°C. The fungal cells were then collected by 3000 *g* and total RNA samples were extracted according to the instructions of MagExtractor-RNA kit (Toyobo, Tokyo, Japan). Six microliters of extracted total RNA was incubated with 2 μL 4× DNA Master I (containing gDNA Remover) and 2 μL 5RT-Master Mix II, and reverse-transcribed into cDNA as recommended by ReverTra Ace qPCR RT Master Mix with gDNA Remover kit (Toyobo, Tokyo, Japan) with procedures as follows: 65°C for 5 min and 4°C for 1 min for initial RNA denaturation, followed by 37°C for 15 min, 50°C for 5 min, and 4°C for 1 min. The prepared cDNA was diluted 10-fold prior to the use for RT-PCR. Primers for *C. albicans* (Table 1) were designed by Primer Premier 5.0 and synthesized by Sangon Biotech (Shanghai, China), while those for *C. tropicalis*, *C. parapsilosis*, *C. krusei*, and *C. glabrata* were originated from the studies published previously (Sanguinetti et al., 2005; Ricardo et al., 2014; Souza et al., 2015; Shi et al., 2017). Twenty-five microliters of real time PCR mixture was freshly prepared containing 12.5 μL of 2× SYBR Green Realtime PCR, 1 μL of PCR Forward Primer, 1 μL of PCR Reverse Primer, 0.5 μL of cDNA, and 10 μL of ddH2O. The PCR process were performed on ABI7000 fluorescent quantitative PCR system (Applied Biosystem) with following cycles: 95°C for 60 s for pre-denaturation alone with 95°C for 15 s, 55°C for 15 s, 72°C for 45 s for a total of 40 cycles. All data were normalized to housekeeping gene ACT1 as the internal reference gene. The relative target-gene expression was calculated as a fold change of 2^−ΔΔCt^ value, in which ΔCt = Ct^targetgene^ - Ct^internal referencegenes^ as previously described (Livak and Schmittgen, 2001).

**Table 1:** Primers for qRT-PCR.

Genes	Forward (5′-3′)	Reverse (5′-3′)
**ACT1**	ACCGAAGCTCCAATGAATCC	CCGGTGGTTCTACCAGAAGAG
***C. albicans* Z3044/SC5314**		
CDR1	TGGTGCCATGACTCCTGCTA	CCATCGAGACCAACCCAACA
CDR2	GCCAATGCTGAACCGACAGA	AGGACCAGCCAATACCCCAC
MDR1	CCACTGGTGGTGCAAGTGTT	GGACCACAAACAGCACCCAA
FLU1	TCCAATGCTTGGTTCACGAGATCC	GGATACCGATAAGGCAGCAAGACC
***C. glabrata* ATCC2340**		
CDR1	TAGCACATCAACTACACGAACGT	AGAGTGAACATTAAGGATGCCATG
CDR2	GTGCTTTATGAAGGCTACCAGATT	TCTTAGGACAGAAGTAACCCATCT
SNQ2	ACCATGTGTTCTGAATCAATCAAT	TCGACATCATTACAATACCAGAAA
***C. parapsilosis* ATCC22019**		
CDR1	ATTTGCCGACATCCACCGTTAGG	ACCATGCTGTTTGCGAGTCCA
MDR1	GATTTTTCGCTAGTCCGTGTTTG	TGTAGGCGCATAGGTCTCAGGT
***C. krusei* ATCC1182**		
ABC1	GATAACCATTTCCCACATTTGAGT	CATATGTTGCCATGTACACTTCTG
ABC2	CCTTTTGTTCAGTGCCAGATTG	GTAACCAGGGACACCAGCAA
***C. tropicalis* GDM 2.147**		
CDR1	TGGAAAGAGTTGGAGGGTATGTTA	TCCCAAGGTTTCGCCATC
MDR1	TTGGCGTTAGAGGATTTACTTTGG	GAATGAAAACTTCTGGGAAAACTGG

### Statistical Analysis

All experiments were performed in triplicate in three individual workdays. The results were recorded as mean ± standard deviation (*n* = 3) and calculated by SPSS 17.0 (SPSS Inc., Chicago, IL, United States). The data among groups were analyzed by one-way ANOVA with least significance difference (LSD) method, in which *p* < 0.05 was considered as statistically significant.

## Results and Discussion

### Synergistic Activity of PAL With FLC and ITR Against Planktonic and Biofilm Candida Cells

In planktonic cells, the susceptibility test showed that the MICs of PAL were in a range of 128–512 μg/mL against *C. albicans* SC5314 and six clinical isolates and 64 ≥ 1024 μg/mL against the five NAC isolates. The MICs of PAL ranged from 0.5 to 64 μg/mL in *C. albicans* isolates and 4 to 128 μg/mL in the five NAC isolates after in combination with FLC compared with the MICs alone (Table 2). Likewise, the MICs of PAL altered between 0.5–16 μg/mL in *C. albicans* isolates and 2–32 μg/mL in the five NAC isolates following simultaneous use of ITR compared with the MICs alone (Table 3). The FICIs were, respectively, of 0.0049–0.75 for PAL plus FLC and 0.0059–0.3125 for PAL plus ITR, reflecting the action mode of PAL+FLC/ITR could be defined as synergism in most isolates tested except *C. albicans* SC5314 and *C. tropicalis* GDM 2.147 in PAL plus FLC (Tables 2, 3).

**Table 2:** Interactions of PAL alone/in combination with FLC and PAL alone/in combination with ITR against planktonic cells of Candida spp.

Strains	MIC_80_ alone (μg/mL)			MIC_80_ in combination (μg/mL)		(Interpretation) FICI of PAL+FLC	(Interpretation) FICI of PAL+ITR
	PAL	FLC	ITR	PAL/FLC	PAL/ITR		
*Candida guilliermondii* ATCC6260	>1024	2	128	128/0.0625	8/2	0.1563 (synergism)	0.0234 (synergism)
*Candida glabrata* ATCC2340	512	2	2	128/0.0625	32/0.125	0.2813 (synergism)	0.2813 (synergism)
*Candida tropicalis* GDM 2.147	64	1	16	4/0.5	2/2	0.5625 (indifference)	0.1563 (synergism)
*Candida krusei* ATCC1182	512	2	1	128/0.125	32/0.125	0.3125 (synergism)	0.1875 (synergism)
*Candida parapsilosis* ATCC22019	64	2	16	4/0.0625	8/0.5	0.0938 (synergism)	0.1563 (synergism)
*Candida albicans* SC5314	128	0.5	4	64/0.125	4/0.125	0.75 (indifference)	0.0625 (synergism)
*Candida albicans* Z3044	128	>1024	4	1/1	2/1	0.0088 (synergism)	0.2656 (synergism)
*Candida albicans* Z2003	128	>1024	8	1/1	16/1	0.0088 (synergism)	0.25 (synergism)
*Candida albicans* Z1402	128	>1024	4	0.5/2	2/0.5	0.0059 (synergism)	0.1406 (synergism)
*Candida albicans* Z1407	512	>1024	4	2/4	8/1	0.0195 (synergism)	0.2656 (synergism)
*Candida albicans* Z826	128	>1024	8	1/1	8/2	0.0088 (synergism)	0.3125 (synergism)
*Candida albicans* Z1309	128	> 1024	8	0.5/1	8/2	0.0049 (synergism)	0.3125 (synergism)

**Table 3:** Interactions of PAL alone/in combination with FLC and PAL alone/in combination with ITR against biofilm cells of Candida spp.

Strains	SMIC_80_ alone (μg/mL)			SMIC_80_ in combination (μg/mL)		(Interpretation) sFICI of PAL+FLC	(Interpretation) sFICI of PAL+ITR
	PAL	FLC	ITR	PAL/FLC	PAL/ITR		
*Candida guilliermondii* ATCC6260	>1024	8	512	32/256	2/64	0.2813 (synergism)	0.127 (synergism)
*Candida glabrata* ATCC2340	>1024	>1024	>1024	256/128	512/512	0.375 (synergism)	1 (indifference)
*Candida tropicalis* GDM 2.147	256	>1024	>1024	8/256	16/32	0.2813 (synergism)	0.0938 (synergism)
*Candida krusei* ATCC1182	>1024	>1024	>1024	8/128	128/4	0.1563 (synergism)	0.1289 (synergism)
*Candida parapsilosis* ATCC22019	256	2	512	32/0.0625	8/128	0.1563 (synergism)	0.2813 (synergism)
*Candida albicans* SC5314	>1024	>1024	>1024	64/256	64/128	0.3125 (synergism)	0.1875 (synergism)
*Candida albicans* Z3044	>1024	>1024	>1024	64/256	16/256	0.3125 (synergism)	0.2656 (synergism)
*Candida albicans* Z2003	>1024	>1024	>1024	32/256	32/128	0.2813 (synergism)	0.1563 (synergism)
*Candida albicans* Z1402	>1024	>1024	>1024	32/256	64/256	0.2813 (synergism)	0.3125 (synergism)
*Candida albicans* Z1407	>1024	>1024	>1024	64/64	32/32	0.125 (synergism)	0.0625 (synergism)
*Candida albicans* Z826	>1024	>1024	>1024	64/256	64/128	0.3125 (synergism)	0.1875 (synergism)
*Candida albicans* Z1309	>1024	>1024	>1024	32/128	32/64	0.1563 (synergism)	0.0938 (synergism)

A number of studies evaluated the MICs of PAL against a series of *Candida* species and found that they were of ≥500 μg/mL against *C. albicans*, *C. glabrata*, *C. tropicalis*, *C. krusei*, and *C. guilliermondii*, except *C. parapsilosis* which was inhibited at 15.6 μg/mL (Iwasa et al., 1998; Park et al., 1999; Vollekova et al., 2003). In this study, the MICs of PAL alone were lower than or approximate to 500 μg/mL against most *Candida* isolates (11/12) aside from *C. guilliermondii* ATCC6260 (MIC > 1024 μg/mL). This discrepancy might be due to the limited purity of PAL that was extracted from effective part of medicinal plants in those previous studies compared with ours. Intriguingly, the presence of PAL improved tremendously the susceptibility of *Candida* species, especially FLC-resistant *C. albicans* isolates, to FLC by even more than 1000-fold increase. Meantime, we also observed that the addition PAL promoted significantly the efficacy of ITR with an elevation of 2- to 64-fold compared with ITR used alone. These results showed the effectiveness of PAL in combination with FLC and ITR to inhibit planktonic cells of *Candida* strains.

The biofilm is a self-protected life-mode of *Candida* species different from the planktonic state with complex structure and powerful resistance to most antimycotics. The formation of biofilm therefore is supposed to be a crucial factor to render the incapability of conventional antifungal agents (Tobudic et al., 2012; Taff et al., 2013). Prior to this study, the anti-biofilm potential of PAL and its synergistic effect with azoles has not been documented to our knowledge. Herein, the SMIC_80_ were above 1024 μg/mL for PAL, FLC, and ITR being used individually in most tested *Candida* species, while decreased significantly by 4- to 128-fold for PAL and 4- to 32-fold for FLC, respectively, with FICI in a range of 0.125–0.375 by PAL plus FLC, and reduced remarkably by 8- to 512-fold for PAL and 8- to 256-fold for ITR, respectively, with FICI ranging from 0.0938 to 0.3125 by PAL plus ITR. The pairing of PAL plus FLC showed synergism for all of the tested strains, and so did that of PAL plus ITR except *C. glabrata* ATCC2340 (Table 3). These results demonstrated that the concomitant uses of PAL and FLC/ITR were applicable in the removal of *Candida* biofilms.

**FIGURE 1:**
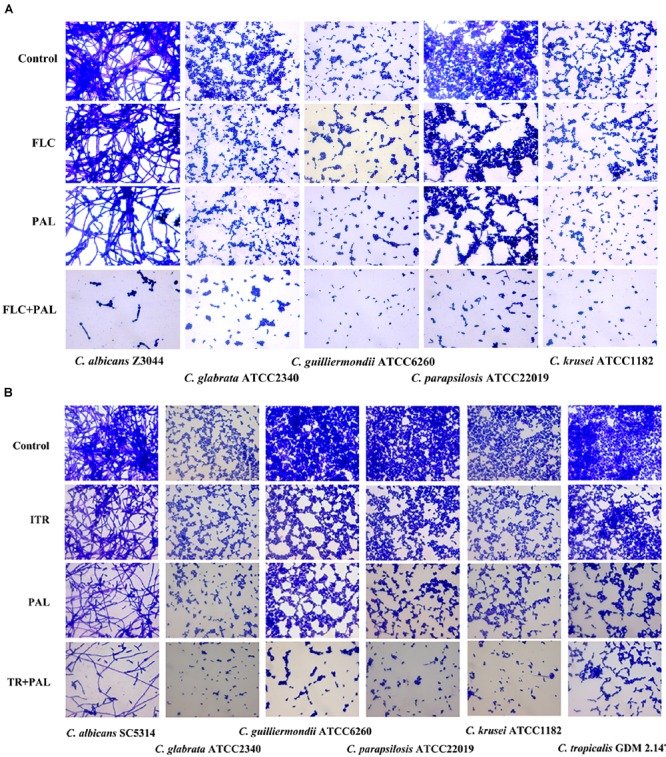
Gram staining of *Candida* species incubated with **(A)** PAL, FLC, PAL+FLC, and **(B)** PAL, ITR, PAL+ITR at the concentrations of their MICs showing synergism. The initial inoculum was newly prepared at 2 × 10^6^ CFU/mL. The drug-free strain-containing medium was set as the control. Oil lens: ×1000.

**FIGURE 2:**
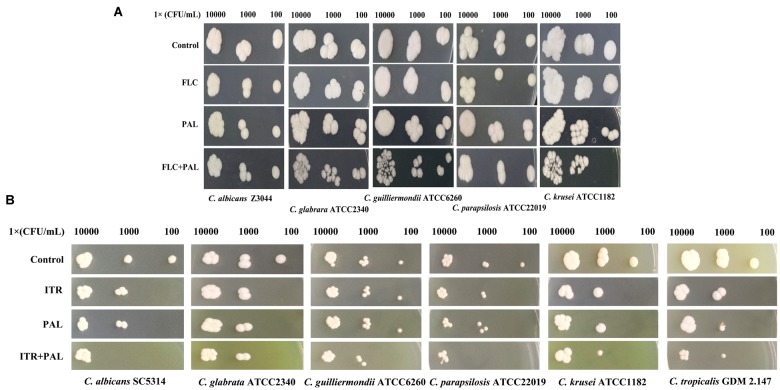
Spot assay of *Candida* species spreading on YPD agar containing **(A)** PAL, FLC, PAL+FLC, and **(B)** PAL, ITR, PAL+ITR at the concentrations of their MICs showing synergism. The initial inoculum was 10-fold diluted in a range of 1 × 10^2^–1 × 10^4^ CFU/mL. The drug-free strain-containing medium was set as the control.

In the following gram staining test and spot assay, the strong synergisms of PAL plus FLC/ITR were also confirmed. Without drug treatment, as shown, quantities of blastoconidia were observed in the tested strains including *C. glabrata* ATCC2340, *C. parapsilosis* ATCC22019, *C. guilliermondii* ATCC6260, *C. krusei* ATCC1182, and *C. tropicalis* GDM 2.147. Apart from *C. krusei* ATCC1182 with very less pseudohyphae, there were absence of hyphal or pseudohyphal forms in the other three isolates. These observations were consistent with the descriptions in several previous literatures, in which it is believed that *C. glabrata* had entirely no presence of pseudohyphae and hyphae, while the other three strains occasionally displayed pseudohyphae in a strain-dependent manner but were deemed to be unable to generate hyphae (Samaranayake and Samaranayake, 1994; Savini et al., 2011; Silva et al., 2012; Rodrigues et al., 2014). In comparison, *C. albicans* exhibited abundant hypha with less yeast cells in drug-free control. Following the treatments of PAL and FLC/ITR, the hyphal and blast conidia cells were remarkably reduced (Figures 1A,B). With 10-fold of series dilution, the combination of PAL with FLC/ITR also demonstrated their strong synergistic effects in the spot assay (Figures 2A,B).

### Inhibition of PAL in Combination With FLC/ITR on Efflux Pumps

**FIGURE 3:**
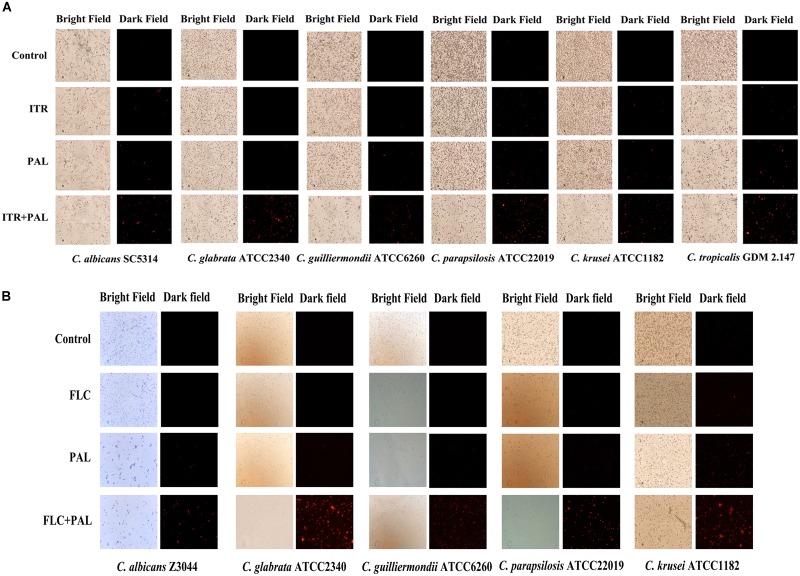
Rhodamine 6G efflux in *Candida* species in incubation with **(A)** PAL, ITR, PAL+ITR, and **(B)** PAL, FLC, PAL+FLC at the concentrations of their MICs showing synergism. The initial inoculum was newly prepared at 2 × 10^6^ CFU/mL. The drug-free strain-containing medium was set as the control. The excitation wavelength and the emission wavelength were of 525 and 550 nm, respectively. The micrographs were recorded in bright field and dark field with a magnification of 200.

The rhodamine 6G is a commonly used fluorescent dye with the same transporters as azoles in yeast (Maesaki et al., 1999). This assay measured the efflux activities in the selected *Candida* species being incubated with PAL and/or FLC/ITR at the concentrations showing synergism in terms of intracellular fluorescence (red color). As shown, drug-free and single drug induced no or less accumulation of intracellular rhodamine 6G, whereas concomitant use of PAL and FLC resulted in vast appearance of red fluorescence inferring there was a close association of the antifungal effects of PAL and/or FLC/ITR with the normal function of efflux pumps in the *Candida* species tested (Figures 3A,B).

**FIGURE 4:**
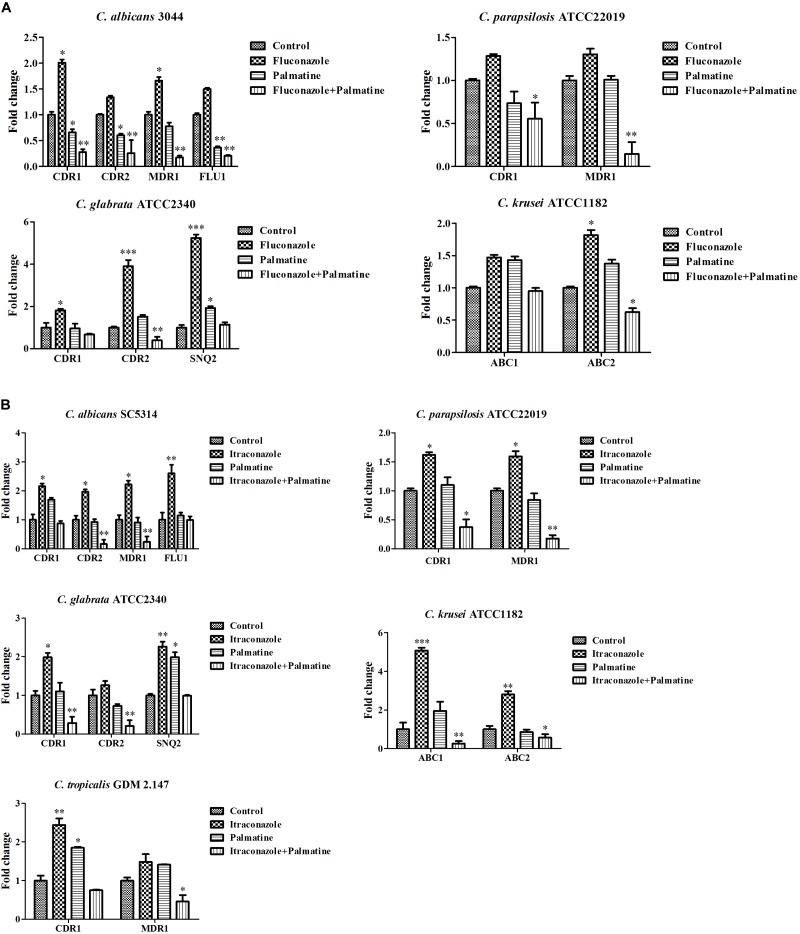
Expressions of efflux-associated genes in *Candida* species in incubation with **(A)** PAL, FLC, PAL+FLC, and **(B)** PAL, ITR, PAL+ITR at the concentrations of their MICs showing synergism. The initial inoculum was newly prepared at 1 × 10^6^ CFU/mL. The drug-free strain-containing medium was set as the control. ^∗^*p* < 0.05; ^∗∗^*p* < 0.01; ^∗∗∗^*p* < 0.001; compared with the control.

It is known that two main classes of efflux pumps are considered to mediate the resistance of *Candida* species, i.e., ABC superfamilies and MFS pumps. The former is driven by ATP hydrolysis, and the latter employs the proton-motive force across the membrane (Cannon et al., 2009). Among the *Candida* species, both transporters are the most extensively studied in *C. albicans*. Current evidence indicated that *CaCDR*1, *CaCDR*2, *CaMDR*1, and *CaFLU*1 had specific substrates of conventional antifungal drugs. Overexpressions of *CaCDR*1 and *CaCDR*2 are correlative to high-frequency resistance in clinical isolates. *CaCDR*1 single mutant was hypersensitive to azoles, and *CDR*1*CDR*2 double mutant became more susceptible to azoles than *CaCDR*1 single mutant (Sanglard et al., 1996, 1997). However, accumulating reports demonstrated that *CaCDR*1 may play a more important role than *CaCDR*2 in azole resistance (Andes et al., 2006a,b; Holmes et al., 2008). Considering significance of *CDR*1 and *CDR*2 versus *MDR*1 and *FLU*1 in the resistance of *C. albicans* to azoles, it should be noted that CaCdr1p and CaCdr2p possess more azole substrates than CaMdr1p which is relatively specific for FLC (Cannon et al., 2009). Of interest, two independent pathways might be possibly present to control the levels of *CDR*1, *CDR*2, and *MDR*1 in azole-resistant *C. albicans* as the expressions of *CDR*1 and *CDR*2 were upregulated in some FLC-resistant strains, while the expression of *MDR*1 was upregulated in other strains (Cannon et al., 2009). The expression of *FLU*1 seemed to be not required in azole resistance of *C. albicans* as *FLU*1 mutant affected FLC susceptibility negligibly. Basically, the association of azole resistance with the levels of ABC superfamilies is comparatively stronger than MFS pumps (Cannon et al., 2009). Our PCR results revealed comparable decreases of *CDR*1 and *CDR*2 and considerable reductions of the four genes after treatments of PAL and PAL plus FLC (Figure 4A). We evaluated the expressions of *CDR*1 and *CDR*2 belonging to ABC superfamilies, *MDR*1 and *FLU*1 members of MFS pumps in a clinical isolate *C. albicans* Z3044 exposed to PAL and/or FLC and the standard reference strain *C. albicans* SC5314 treated with PAL and/or ITR. The results showed that the use of PAL alone with FLC inhibited the expressions of the four genes significantly (*p* < 0.05, Figure 4A), while the use of PAL alone with ITR only restrained the expressions of *CDR*2 and *MDR*1 (*p* < 0.01, *p* < 0.05, Figure 4B). It is speculated that the contributions of *CDR*1, *CDR*2, *MDR*1, and *FLU*1 to azole resistance are dependent on many factors including at least incubation conditions and strain types in *C. albicans*.

We further evaluated the combined effects of PAL and/or FLC/ITR on the expressions of several efflux-associated genes in *C. parapsilosis* ATCC22019, *C. glabrata* ATCC2340, *C. krusei* ATCC1182, and *C. tropicalis* GDM2.147. Our results revealed that (i) PAL plus FLC/ITR caused a great impact on *CDR*1 and *MDR*1 (*p* < 0.05, *p* < 0.01) in *C. parapsilosis* ATCC22019; (ii) PAL plus FLC only affected the expressions of *CDR*2 (*p* < 0.01), while PAL plus ITR decreased the expressions of *CDR*1 and *CDR*2 dramatically (*p* < 0.01, *p* < 0.01) in *C. glabrata* ATCC2340; (iii) the expressions of *ABC*1 and *ABC*2 were lowered (*p* < 0.01, *p* < 0.05) after the use of PAL+ITR, while only *ABC*2 were suppressed (*p* < 0.05) in the case of PAL+FLC in *C. krusei* ATCC1182; (iv) the combination of PAL and ITR only influenced *MDR*1 (*p* < 0.05) in *C. tropicalis* GDM2.147 (Figures 4A,B).

[In NAC species, ABC superfamilies and MFS pumps are still the most studied efflux pumps in azole resistance, but different from those in *C. albicans* in types and contributions. The comprehensive knowledge of efflux pumps in NAC species is summarized in several literatures (Cannon et al., 2009; Whaley et al., 2017). In a study of elucidating FLC-resistance mechanism in C. *parapsilosis*, *CDR*1 and *MDR*1 were found to be overexpressed in 16 FLC-resistant isolates and 3 other resistant ones, respectively (Berkow et al., 2015). Several reports also observed the close relationship of *CDR*1 and *MDR*1 with FLC-resistance (Grossman et al., 2015; Souza et al., 2015). However, it was also supposed that overexpressions of *CDR*1 and *MDR*1 might not be the sole reason responsible for FLC-resistance in C. *parapsilosis* (Berkow et al., 2015). In *C. glabrata*, there are three ABC transporters identified to be linked directly to azole resistance, namely *CDR*1, *CDR*2 (*PDH*1) and *SNQ*2 (Sanglard et al., 1999, 2001; Torelli et al., 2008). Whereas MFS transporters might exert much less impact on azole resistance in *C. glabrata* (Costa et al., 2016). *C. krusei* has an inherent instinct of resistance to FLC. This innate resistance mechanism might be connected with ATP-binding cassette transporter Abc1p (Katiyar and Edlind, 2001; Lamping et al., 2009). In addition, resistance to ITR was attributed to reduced intracellular drug content and the overexpression of efflux pump Abc2p (Ricardo et al., 2014; He et al., 2015). As with *C. tropicalis*, the expressions of *CDR*1 and *MDR*1 were remarkably upregulated in azole-resistant strains (Barchiesi et al., 2000). We also found a critical role of *CDR*1 and *MDR*1 in berberine-treated *C. tropicalis* isolates (Shi et al., 2017). In our study, accumulated intracellular rhodamine 6G was an intense sign of the involvement of efflux pumps (Figures 3A,B). Subsequent qRT-PCR analyses confirmed the close relationship of most efflux-associated genes tested with the synergistic antifungal mechanisms of PAL plus FLC/ITR due to their varying degrees of inhibition (Figures 4A,B). Nevertheless, it should be noted that azole resistance is a multifactorial event with more than one gene implicated (such as *ERG*11), and more studies are necessarily performed to elucidate the involvements of these genes used in azole resistance and cross-resistance.

### Contributions of Medicinal Plant Drugs and Antifungal Strategy of Combination

Growing evidence exhibit the huge antifungal potential of quantities of medicinal plant drugs which are easily accessible and less toxic to human with decent antifungal activities and reasonable prices (Li et al., 2013; da Silva et al., 2014; Singh et al., 2015; Sun et al., 2015; Shao et al., 2017). Aside from a few herbal drugs showing relatively low MIC alone (such as berberine and sodium houttuyfonate ≤64 μg/mL), the anti-*Candida* effect is relatively weak in a majority of plant drugs (usually MIC > 256 μg/mL). The case is also not changed for other non-conventional antifungal agents which are converted to antifungal purpose (Zhou et al., 2012; da Silva et al., 2013; Letscher-Bru et al., 2013; Shahzad et al., 2014). To strengthen the efficacy of individual drug for antifungal usage and traditional antimycotics in the treatment of *Candida* resistant strains, the combination strategy is a reliable and feasible approach to inspire the antifungal potentials of the two drugs used which can dramatically lower the MIC of each one. Further, the combination method is also a widely used and effective way in the removal of *Candida* biofilms. Lu et al. (2017) made a deep and all-sided review on this field. This study is another successful example of drug combination, by which the relative high MICs and SMICs of PAL and FLC alone were dramatically reduced.

## Conclusion

We confirmed the antifungal effects of PAL alone and in combination with FLC/ITR on planktonic and biofilm cells of *C. albicans* as well as NAC species. The gram staining and spot assay results further demonstrated PAL was intensely synergistic with FLC/ITR against most of the tested strains. The rhodamine 6G and PCR results inferred that there might be a close association of the antifungal mechanism of PAL plus FLC/ITR with efflux pumps. This study indicated PAL might be a promising synergist against cross-resistance to FLC and ITR in *Candida* strains.

## Author Contributions

TW, WD, QL, and GS performed the experiments. DW reviewed the whole manuscript. JS and CW devised the experiments. JS wrote most of the main text. JS, WD, and QL also together arranged the Tables and Figures.

## Conflict of Interest Statement

The authors declare that the research was conducted in the absence of any commercial or financial relationships that could be construed as a potential conflict of interest.
